# The diversity in antimicrobial resistance of MDR *Enterobacteriaceae* among Chinese broiler and laying farms and two *mcr-1* positive plasmids revealed their resistance-transmission risk

**DOI:** 10.3389/fmicb.2022.912652

**Published:** 2022-08-04

**Authors:** Shuaizhou Zong, Dingting Xu, Xiner Zheng, Davood Zaeim, Peng Wang, Jianzhong Han, Daofeng Qu

**Affiliations:** ^1^Key Laboratory of Food Quality and Safety, School of Food Science and Biotechnology, Zhejiang Gongshang University, Hangzhou, China; ^2^The Second Affiliated Hospital, School of Medicine, Zhejiang University, Hangzhou, China

**Keywords:** metagenomic, ARGs, MGEs, multidrug-resistant *Enterobacteriaceae*, poultry farms, *mcr-1*

## Abstract

This research aimed to investigate the microbial composition and diversity of antimicrobial resistance genes (ARGs) found in Chinese broiler and layer family poultry farms. We focused on the differences in resistance phenotypes and genotypes of multidrug-resistant *Enterobacteriaceae* (MDRE) isolated from the two farming environments and the existence and transmissibility of colistin resistance gene *mcr-1*. Metagenomic analysis showed that *Firmicutes* and *Bacteroides* were the dominant bacteria in broiler and layer farms. Many aminoglycoside and tetracycline resistance genes were accumulated in these environments, and their absolute abundance was higher in broiler than in layer farms. A total of 526 MDRE were isolated with a similar distribution in both farms. The results of the K-B test showed that the resistance rate to seven antimicrobials including polymyxin B and meropenem in broiler poultry farms was significantly higher than that in layer poultry farms (*P* ≤ 0.05). PCR screening results revealed that the detection rates of *mcr-1*, *aph(3’)Ia*, *aad*A2, *bla*_*oxa–1*_, *bla*_*CTX–M*_, *fos*B, *qnr*D, *sul1*, *tet*A, and *cat*A1 in broiler source MDRE were significantly higher than those in layers (*P* ≤0.05). A chimeric plasmid p20432-*mcr* which carried the novel integron In1866 was isolated from broiler source MDRE. The high frequency of conjugation (10^–1^ to 10^–3^) and a wide range of hosts made p20432-*mcr* likely to play an essential role in the high detection rate of *mcr-1*, *aph(3’)-Ia*, and *aad*A2 in broiler farms. These findings will help optimize disinfection and improve antimicrobial-resistant bacteria surveillance programs in poultry farms, especially broilers.

## Introduction

Antimicrobial resistance has been characterized as a global public health concern, catalyzed by the over-use of antimicrobials used in farming food-producing animals (Winglee et al., 2017; KöCk et al., 2018). Tons of antimicrobials are used annually as treatments and growth promoters in livestock and poultry farms (Udikovic-Kolic et al., 2014; Xiong et al., 2018). It led to the accumulation of antimicrobials in the farming environments, creating the constant selective pressure for antimicrobial resistance to microorganisms (Udikovic-Kolic et al., 2014), and making poultry a reservoir for resistant bacteria and genes (Mceachran et al., 2015). China has the largest layer chicken industry globally and is the second-largest producer of broiler chickens (Gaze et al., 2013; FAO, 2019). In addition to the large-scale poultry farms, family poultry farms are a typical farming pattern with Chinese characteristics. Especially in rural areas, residential buildings and poultry sheds are often housed in the same yard, facilitating poultry management for farmers. More importantly, it reduces the space needed for independent farms and lowers the threshold for poultry farming. Because of the compact space and frequent contact, farmers face a higher risk of antimicrobial resistance in such family farms. These industries cause massive pollution to the environment by animal excretion. Family farming puts people in close contact with breeding environments, making it easier for antimicrobial-resistant bacteria to spread to people and threaten human health (Wei et al., 2013). The prevalence of resistant bacteria often isolated from feces turn farms into the primary sources of spreading resistant bacteria (Rawat et al., 2018). ARGs are mainly derived from the *Enterobacteriaceae*, which have become increasingly common in human and animal infections (Falagas et al., 2011). Polymyxin is generally considered the last strategy for treating multidrug-resistant *Enterobacteriaceae* (MDRE) (Wang et al., 2017). Although the application of colistin as a growth-promotor in livestock and poultry production has been banned in China (Shi et al., 2020), the prevalence of colistin resistance genes and increasing rates of MDRE (Buelow et al., 2017) in poultry products remains a significant risk to public health that may jeopardize the treatment of serious bacterial infections (Belluco et al., 2016; Zhai et al., 2020).

Most studies on antimicrobial resistance in poultry production have focused on antimicrobial residues and specific ARGs, and few studies have compared broilers and layers (Zhai et al., 2020). There is a gap in understanding the differences in bacterial community structure, antimicrobial resistome, and mobile genetic elements (MGEs) in layer and broiler chicken environments. It is crucial to assess resistance-transmission risk in family poultry farms carefully.

Previous studies on different poultry (broilers and layers) farms suggest that robust screening of antimicrobials leads to the spreading of antimicrobial resistance genes (ARGs) and entering the human food chain (Su et al., 2015). Therefore, the transmission of resistant genes and their hosts in the farm environment needs to be monitored more frequently. This study aimed to analyze the abundance of ARGs and their potential bacterial hosts in the microbial communities in broilers and layers breeding environments and explore the ARGs’ potential transmission risks in breeding poultry and human, as determined by metagenomics sequencing combining analysis of *mcr-1* positive MGEs. These findings will provide a more comprehensive and accurate theoretical basis for risk assessment of antimicrobial resistance in family poultry patterns in China and control further spread of antimicrobial resistance and protect farmers.

## Materials and methods

### Study area and sample collection

Six broilers and layers poultry farms were selected for a comparative study. All farms were located in Jiaxing, Zhejiang Province. All the six environments were small family farms with chicken houses, which is very common in local areas. Each farm had four to six individual chicken houses and each house contained ∼80 chickens. Twenty fecal samples were randomly collected from each chicken house by swab. A total of 150 fecal samples were collected from broiler farms (BFs), and 150 fecal samples were collected from layer farms (LFs) in September 2021. In addition, soil (*n* = 3), water (*n* = 3), and feces (*n* = 3) samples were collected from broiler farms and mixed into a composite sample. The same sampling scheme was also used for layer farms. Two composite samples were used for metagenomic analysis. All samples were transported to the laboratory in normal saline within 8 h to isolate *Enterobacteriaceae*, and each sample enriched in 10 mL of Buffered Peptone Water (BPW, Macklin, China) and incubated overnight at 37°C. MacConkey (MAC) agar (Macklin, China) was used to cultivate *Enterobacteriaceae* strains. A suspected colony of each microorganism was isolated on a slope agar and PCR was used for subsequent identification.

### Metagenomic analysis

Metagenomic sequencing was performed using Illumina Hiseq 4000 with the sequencing strategy of Index 250 PE (paired-end sequencing). Raw data is filtered using fastp (Chen et al., 2018) to correct base and quality-filtered. Low-quality reads were filtered to ensure that (1) the reads were aligned to proper adaptors or primers, (2) the reads contained <10% unknown bases, (3) the reads contained >50% high-quality bases (2013). Bowtie2 (Langmead and Salzberg, 2012) was used to remove reads from the host genome or contamination with high similarity. On average, 9.5 GB of clean reads were generated for each sample. ARG-OAP PROTO (Yin et al., 2018), an analysis pipeline that integrates two common resistance gene databases, including ARDB (Liu and Pop, 2009) and CARD (Alcock et al., 2019), identifies assembly sequences based on the hidden Markov model (HMM) was used to characterize the profile and quantify the relative abundance of ARGs.

### Antimicrobial susceptibility test

The antimicrobial susceptibility of isolated bacteria was tested with the broth dilution method which is recommended by the Clinical and Laboratory Standards Institute (Institute CLSI, 2020) on Mueller-Hinton (MH) broth and evaluated based on the breakpoint and classified as resistant, intermediate, or susceptible (the breakpoints were shown in Supplementary Table 1). The strains exhibiting resistance to three or more classes of antimicrobials were considered to be multidrug-resistant organisms.

### PCR amplification and sequencing of 16S rRNA

The isolated bacteria were identified by VITEK-2 Compact and 16S rRNA gene identification using the universal 16S primers 27F and 1492R (Lane, 1991). The system amplified by 5 μL PCR was added into 1.2% agarose gel for electrophoresis, and the voltage was set at 120 V for 30 min. PCR amplification consisted of denaturation at 96°C for 5 min followed by denaturation at 95°C for 30 s, annealing at 50°C for 90 s, and polymerization at 72°C for 60 s for a total of 30 cycles, and a final extension at 72°C for 5 min. A Western blot detection system was used to observe whether agarose gel had corresponding bands at 1500 bp after electrophoresis. PCR product of successful amplification was used paired-end sequencing (2 × 150 bp) by Sangon Biotech (Shanghai, China).

### PCR amplification and sequencing of antimicrobial resistance genes

The isolated bacteria were screened for nine major classes of antimicrobial resistance genes usually used for human clinical treatment or poultry by PCR using the primers listed in Supplementary Table 2. PCR amplification was carried out as follow: initial denaturation at 95°C for 5 min, denaturation at 94°C for 30 s, annealing at their respective annealing temperature for 30 s, and polymerization at 72°C for 40 s for a total of 30 cycles, and a final extension at 27°C for 10 min. All the PCR products were subjected to Sanger sequencing.

### Conjugation experiments

To access the transferability of *mcr-1* positive plasmids, conjugal transfer experiments were performed with rifampin-resistant *E. coli* strain EC600 used as a recipient and each of the *mcr-1* positive RB20432 and DB20D28 isolates as a donor. The donor was cultured on Brain Heart Infusion (BHI) agar plates with 2 μg/ml polymyxin, and the recipient *E. coli* strain EC 600 was cultivated on BHI agar plates with 2.5 mg/mL rifampicin. After incubation at 37 C for 24 h, colonies growing on selective plates were further confirmed by antimicrobial sensitivity experiments and VITEK-2 Compact.

### Sequencing and analysis of plasmids

The two conjugative plasmids of the transconjugants RB20432-EC600 and DB20D28-EC600 were sequenced by the Illumina HiSeq platforms. The two plasmid DNA was extracted from the transconjugants RB20432-EC600 and DB20D28-EC600 using the SanPrep Column Plasmid Mini-Preps Kit. After extraction, plasmid sequencing was performed with a pair-end library with an average insert size of 300 bp on a Hiseq sequencer (Illumina). Quality control and removing low-quality data were performed with Trim Momatic 0.36 (Bolger et al., 2014). Gapcloser (Nederbragt, 2014) was used to fill gaps. Cytoscape (Smoot et al., 2011) was used to spline the sequence to obtain the final cyclized plasmids. The plasmid sequences were submitted to Rast (Aziz et al., 2008), Genemarks, Glimmer, and Prodigal library for preliminary gene prediction then submitted to ISFinder, Tn Number Registry, and INTEGRALL (Moura et al., 2009) for MGEs.

### Statistical analysis

The data of bacterial and ARGs were processed with Microsoft Excel 2016. The column was generated using Origin 2021. The heat map for the resistance profile of MDRE was visualized *via* Heatmapper^1^ (Sasha et al., 2016). Antimicrobial susceptibility and resistance gene detection of MDRE isolates were analyzed by independent sample *t*-tests and SPSS ver.21, and *P* ≤ 0.05 was considered statistically significant. Running a gene alignment program in Perl language with Ubuntu 18.04 LTS1 and Inkscape 0.48.1 were used to draw gene organization diagrams.

## Results

### Characterization of bacterial and antimicrobial resistance gene communities in layer farms and broiler farms

**FIGURE 1:**
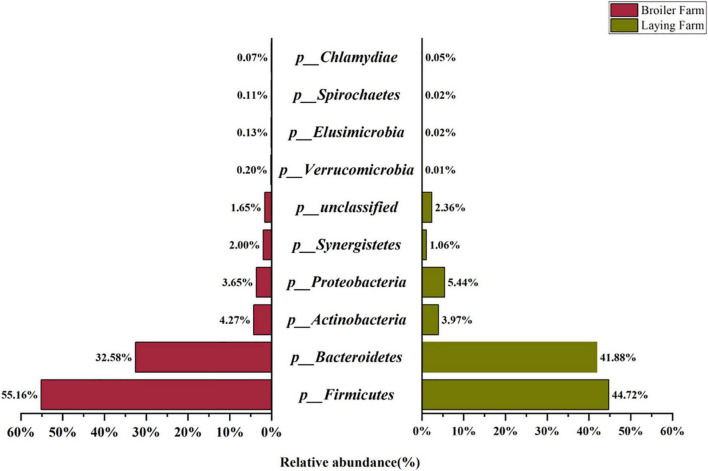
Top ten relative abundance of taxonomic composition at the phylum level in broiler and laying farm samples.

The top ten relative abundance of the microbial communities at the phylum levels are shown in Figure 1. *Firmicutes* and *Bacteroidetes* were the top two in all bacteria phyla, with 55.16% and 32.58% in the BF samples, 44.72% and 41.88% in the LF samples. It can be inferred that *Firmicutes* and *Bacteroidetes* are the dominant bacteria in broiler and layer breeding environments, which were potential hosts for ARGs. *Actinobacteria, Proteobacteria*, and *Synergistetes* were the second dominant phyla.

**FIGURE 2:**
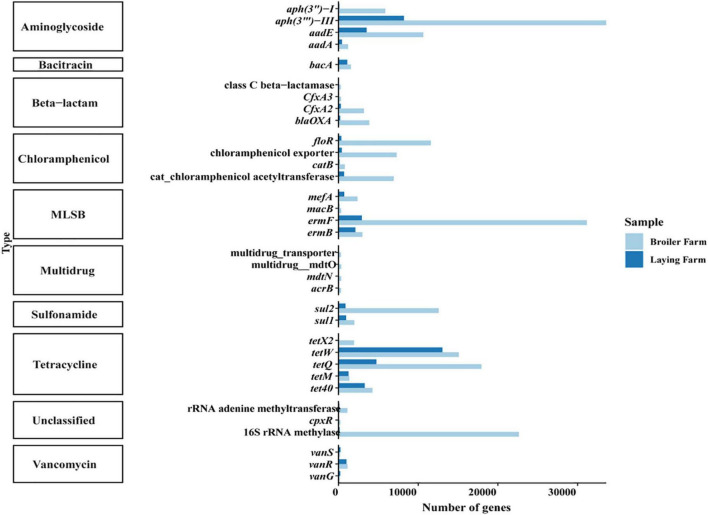
The absolute abundance of top ten ARG classification and their subtypes.

The top ten antimicrobial resistance genes in broiler and laying farms in absolute abundance were aminoglycosides, bacitracin, β-lactam, chloramphenicol, MLSBs (macrolide-lincosamide-streptomycin B), multidrug resistance genes, sulfonamides, tetracyclines, vancomycin and unclassified ARGs (Figure 2). ARGs of all ten classifications were generally detected in broilers, while *MDR_transporter* and *cpx*R were not detected in layers. Many aminoglycoside and tetracycline resistance genes were accumulated in broilers and layers, and their absolute abundance in broilers was higher than that in layers. In broiler samples, *aph(3″′)-III* had the highest absolute abundance, followed by *erm*F, 16S rRNA methylase, and *tet*Q. The absolute abundance of *tet*W in the laying samples was the highest, followed by *aph(3″′)-III*, *tet*Q, and *aad*E. The absolute abundance of vancomycin resistance genes *van*G and *van*S in the laying samples was higher than in broiler samples.

### Detection of multidrug-resistant *Enterobacteriaceae* in layer farms and broiler farms

**FIGURE 3:**
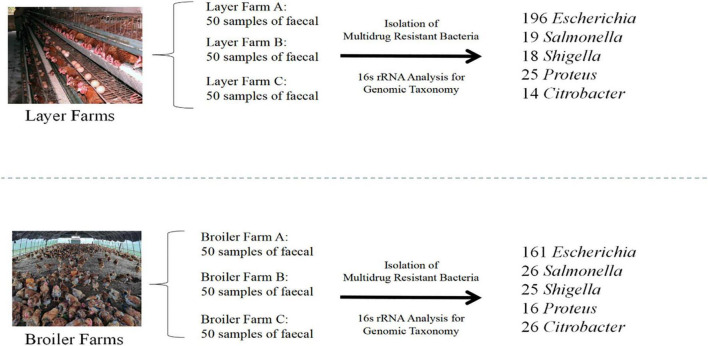
Collection and isolation of MDR *Enterobacter.*

Five hundred twenty-six MDR *Enterobacteriaceae* were recovered from 300 fresh fecal samples, 272 in BFs and 254 in LFs. According to the general prevalence of MDRE layers and broilers, *E. coli*, whose percent reached 67.8% (357/526), was the dominant bacteria. The rest were *Salmonella* (8.6%, 45/526), *Shigella* (8.2%, 43/526), *Proteus* (7.8%, 41/526), and *Citrobacter* (7.6%, 40/526) (Figure 3).

### Differences in antimicrobial-resistant phenotypes of MDRE in broiler farms and layer farms

**FIGURE 4:**
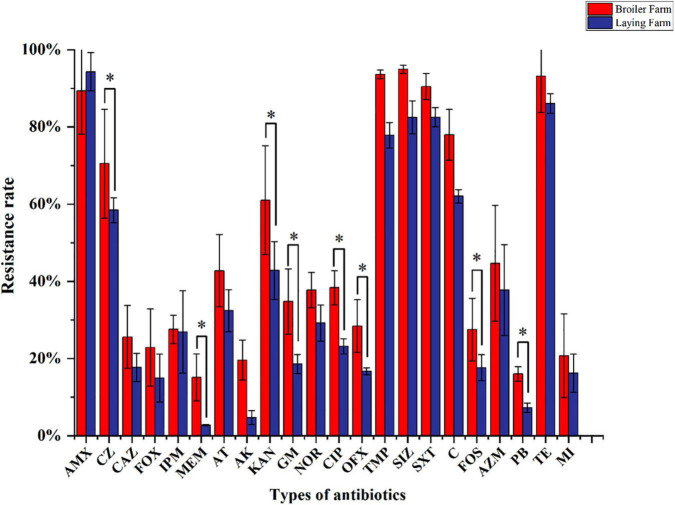
Resistance rates of MDRE isolated from broilers and layers to 25 antimicrobials. AMX, Amoxicillin; CZ, Cefazolin; CAZ, Ceftazidime; FOX, Cefoxitin; IPM, Imipenem; MEM, Meropenem; AT, Aztreonam; AK, Amikacin; KAN, Kanamycin; GM, Gentamicin; CIP, Ciprofloxacin; NOR, Norfloxacin; OFX, Ofloxacin; TMP, Trimethoprim; SIZ, Sulfamethoxazole; SXT, Trimethoprim-Sulfamethoxazole; C, Chloramphenicol; FOS, Fosfomycin; AZM, Azithromycin; PB, Polymyxin B; TE, Tetracycline; MI, Minocycline.

MDRE isolated from 6 farms showed different resistance rates to 25 antimicrobials (Figure 4). Amoxicillin had the highest resistance rate among the 25 antimicrobials, 89.33% in BFs and 94.3% in LFs. In addition, MDRE in BFs and LFs showed more than 80% resistance rates to five antimicrobials: Amoxicillin, sulfamethoxazole, trimethoprim-sulfamethoxazole, and tetracycline. Comparison between the two farms showed that resistance rates of polymyxin B, meropenem, amikacin, gentamicin, ciprofloxacin, sulfamethoxazole, and chloramphenicol were significantly higher in BFs than in LFs (*P* ≤ 0.05). Polymyxin B, banned in poultry production in recent years, has a high resistance rate in BFs, indicating that its resistance gene has been widely spread in the broiler environment.

### Antimicrobial resistance of *Enterobacteriaceae* in broilers was more severe than in layers

**FIGURE 5:**
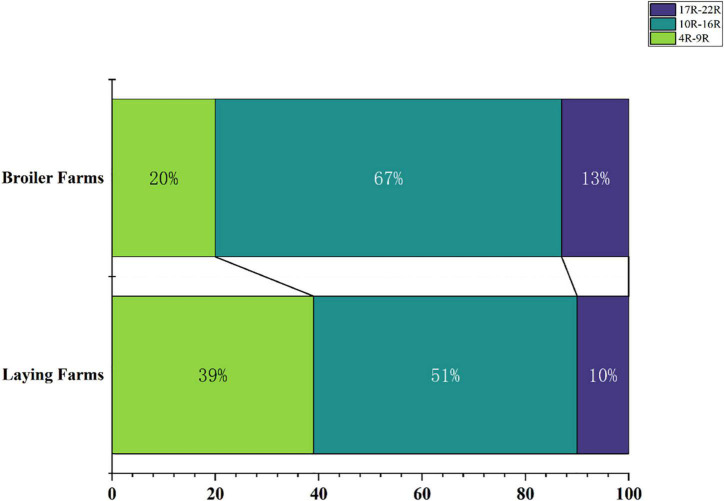
Proportion of resistant bacteria in different drug resistance situations in broiler farms and laying farms.

**FIGURE 6:**
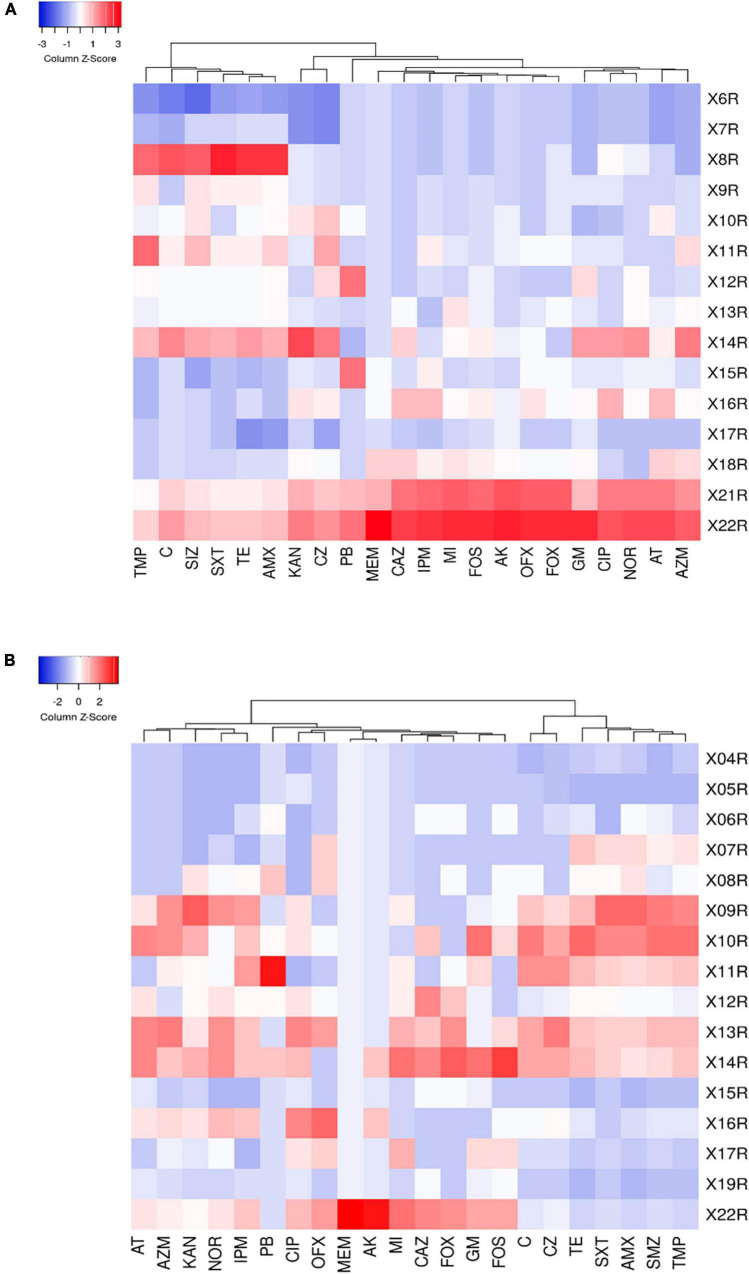
**(A)** Heat map of multidrug-resistance of resistant strains in broiler farms. **(B)** Heat map of multidrug-resistance of resistant strains in laying farms. The right column shows the number of antimicrobials which were resisted by MDRE.

To reflect the multidrug-resistance profile of MDRE in the culture environment more directly, they were divided into three resistance levels: mild antimicrobial resistance (4R∼9R, the number represents the variety of antimicrobials, and “R” represents “Resistance”), moderate antimicrobial resistance (10R∼16R), and severe antimicrobial resistance (17R∼22R) according to the variety of antimicrobials which MDRE was resistance to Figure 5. The proportion of moderate and severe resistance MDRE in BFs was higher than LFs, indicating that the MDR profile of *Enterobacteriaceae* in BFs was more severe than LFs. Most of the MDRE in both LFs and BFs were at the level of moderate antimicrobial resistance, based on which the resistance spectrum of BFs and LFs was deduced. The MDRE in BFs were mainly resistant to ciprofloxacin, cefazolin, kanamycin, minocycline, polymyxin B, and trimethoprim (Figure 6A). However, the heatmaps showed that the MDRE in LFs were most resistant to ofloxacin, fosfomycin, aztreonam, ceftazidime, polymyxin B, and tetracycline (Figure 6B). It should be noted that most 11R and 12R MDRE which have a large base in LFs and BFs were resistant to polymyxin B. Severe antimicrobial resistance MDRE especially 21R and 22R in BFs, was resistant to polymyxin B, meropenem, and imipenem, which should be monitored more frequently. In addition, tetracycline, macrolides, sulfonamides in folic acid metabolic pathway inhibitors, and amoxicillin in β-lactam were resistant; these antimicrobials should be avoided in poultry.

### Differences in antimicrobial resistance genes detection between broiler and layer farms

To investigate the prevalence of ARGs in the poultry environment, resistance genes of nine categories of antimicrobials were detected by PCR analysis. The detection rates of ARGs, *bla*_*TEM*_ (β-lactamases), *tet*A (tetracycline), *sul1* (sulfonamides), *flo*R (chloramphenicol), *fos*A3 (fosfomycin), *mph*(A) (macrolides), *mcr-1* (polymyxins), *qnr*S (quinolones) and *aac(6′)-Ib- cr* (aminoglycoside), were significantly higher than the other ARGs in the same category (*P* ≤ 0.05). The detection rates of each gene are detailed in Supplementary Data 1 and Supplementary Figure 1. In addition, we focused on antimicrobial resistance genes that are banned in poultry production and used differently in layers and broilers for subsequent analysis. Some of these antimicrobials, such as carbapenems and polymyxin, are only available for clinical use but not for use in animals. To avoid antimicrobial residues in eggs, some antimicrobials are banned during the laying period, such as tetracycline, quinolones (danofloxacin, pefloxacin, norfloxacin, ciprofloxacin), macrolides (kitasamycin), β-lactam (amoxicillin, ampicillin), sulfonamides (sulfachlorpyridazine sodium), polymyxin (polymyxin B).

**FIGURE 7:**
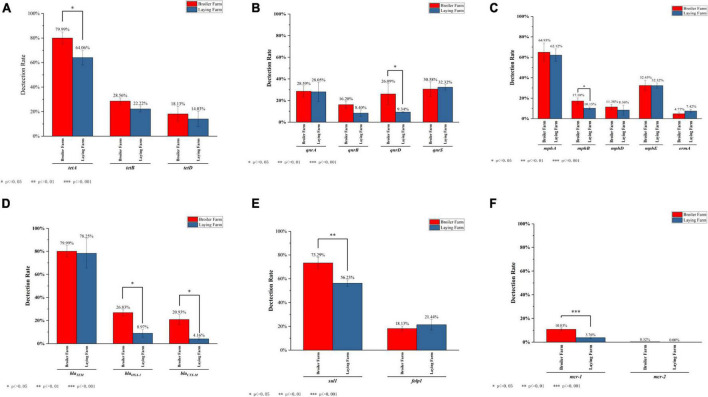
The detection rate of ARGs which are banned in layers or both. **(A)** The detection rate of tetracyclines resistance genes. **(B)** The detection rate of quinolones resistance genes. **(C)** The detection rate of macrolides resistance genes. **(D)** The detection rate of β-lactam resistance genes. **(E)** The detection rate of sulfonamide resistance genes. **(F)** The detection rate of polymyxins resistance genes.

Three kinds of tetracycline resistance genes were detected, and the detection rates of *tet*A were all above 60% in LFs and BFs. The detection rate of *tet*A in BFs was significantly higher than in LFs (*P* ≤ 0.05). Although *tet*D was detected in both LFs and BFs, there was no significant (*P* > 0.05) difference (Figure 7A). MDRE containing quinolone resistance genes *qnr*A, *qnr*B, *qnr*D, *qnr*S were detected in LFs and BFs. There is a significant difference in the detection rates of *qnr*D between BFs and LFs (*P* ≤ 0.05), and the detection rates of *qnr*A, *qnr*B, and *qnr*D were all higher in BFs. In contrast, the detection rate of *qnr*S was higher in LFs than in BFs (Figure 7B). Among the macrolide resistance genes, the detection rate of *mph*(A) was the highest in broiler and layer farms, both exceeding 60% (Figure 7C). The detection rates from high to low were *mph*(A), *mph*(E), *mph*(B), *mph*(D), *erm*A, respectively, and other macrolide resistance genes were not detected. The detection rate of *mph*(B) in BFs was significantly higher (*P* ≤ 0.05). Only three β-lactam resistance genes were identified: *bla*_*TEM*_, *bla*_*OXA–1*_, and *bla*_*CTX–M*_. Except that *bla*_*TEM*_ had high detection rates in both broiler and layer farms, the detection rates of *bla*_*OXA–1*_ and *bla*_*CTX–M*_ in broiler farms were significantly higher than those in LFs (*P* ≤ 0.05) (Figure 7D). Among the sulfonamides resistance genes, only *sul1* and *folp1* were amplified in MDRE. The total detection rate of *sul1* was higher than *folp1* in BFs and LFs (Figure 7E). Moreover, the detection rate of *sul1* in BFs was significantly higher than that in LFs (*P* ≤ 0.01). The detection rate of polymyxin resistance gene in broiler and layer farms was not high, and *mcr-1* was detected in both farms and was significantly higher in BFs than in LFs (*P* ≤ 0.01) (Figure 7F). However, *mcr-2* was only detected in BFs, and the detection rate was 0.32%.

### The *mcr-1* positive plasmids p20432-*mcr* and p2021-*mcr* were conjugative

The transconjugants isolated by mating RB20432 and DB20D28 strains with EC600 were resistant to rifampicin and polymyxin antimicrobials, possibly indicating plasmids transfer. Experiments showed that polymyxin resistance could be successfully transferred from RB20432 and DB20DB to EC600 at 10^–1^ to 10^–3^ (p20432-*mcr*) and 10^–2^ to 10^–3^ (p2021-*mcr*) cell per recipient cell by conjugation. As shown in the table (Table 1), the minimum inhibitory concentrations for polymyxin B for the transconjugant increased 16-fold compared with the untransformed control. In addition, the minimum inhibitory concentration for ampicillin (32-fold), amoxicillin (32-fold), tetracycline (32-fold), gentamicin (eightfold, only RB20432), and sulfamethoxazole (16-fold) were increased to different degrees, suggesting that resistance genes of these antimicrobials also existed in p20432-*mcr* and p2021-*mc*r and were transferred through conjugation.

**TABLE 1:** The result of MIC by using VITEK-2.

Category	Antimicrobial agent			MIC (mg/L)/Antimicrobial susceptibility		
				
		EC600	RB20432	RB20432-*mcr-1*-EC600	DB20D28	DB20D28-*mcr-1*-EC600
Penicillin	Ampicillin	8/S	≥256/R	≥256/R	≥256/R	≥256/R
	Amoxicillin	≤4/S	≥256/R	≥256/R	≥256/R	≥256/R
β-lactam	Ceftazidime	≤4/S	≤4/S	≤4/S	≤4/S	≤4/S
	Imipenem	≤1/S	≥16/R	≤1/S	≤1/S	≤1/S
Colistin	Polymyxin B	≤0.5/ S	≥8/R	≥8/R	4/I	≥8/R
Tetracycline	Tetracycline	4/S	≥128/R	≥128/R	≥128/R	≥128/R
Aminoglycosides	Kanamycin	8/S	≥128/R	32/I	≥64/R	≥64/R
	Azithromycin	8/S	≥128/R	≥64/R	≤4/S	≤4/S
	Gentamicin	≤1/S	≥16/R	8/I	≤1/S	≤1/S
Quinolines	Ciprofloxacin	≤0.25/S	≥4/R	≥4/R	≤0.25/S	≤0.25/S
Sulfonamides	Sulfamethoxazole	≤20/S	≥320/R	≥320/R	≥320/R	≥320/R

S, susceptible; R, resistant; I, intermediate.

### Characterization of multidrug-resistant plasmids p20432-*mcr* and p2021-*mcr*

**FIGURE 8:**
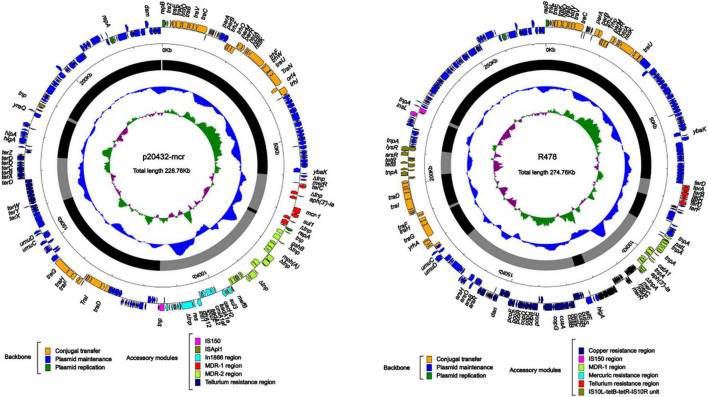
The complete sequence circular diagram of p20432-*mcr* and R478. The innermost ring represents (G–C)/(G + C); the blue ring indicates GC content, and the outward depression indicates that the GC content is higher than the mean value. Black area in the outer circle represents the backbone area and gray area represents the accessory modules. The outermost circle shows the distribution of genes represented by colored arrows in the plasmid.

Two *mcr-1* positive multidrug-resistant plasmids p20432-*mcr* and p2021-*mcr* were found in the polymyxin-resistant MDRE, isolated from layers and broilers fecal samples, respectively. As shown in Figure 8, the total length of plasmid 20432-*mcr* was 228.7 kb, containing 275 ORFs. Furthermore, it had a complex region with two replicons (IncHI2 and IncN), and the backbone was interrupted by accessory modules. The content of GC was 47.3%, and the plasmid contained four accessory modules which were MDR-1 region, MDR-2 region, In*1866*-related region, and metal resistant region. The single replicon IncN plasmid R46 (GenBank: AY046276.1) was chosen as the reference plasmid with similar *rep*A, intI1, and resistance genes.

**FIGURE 9:**
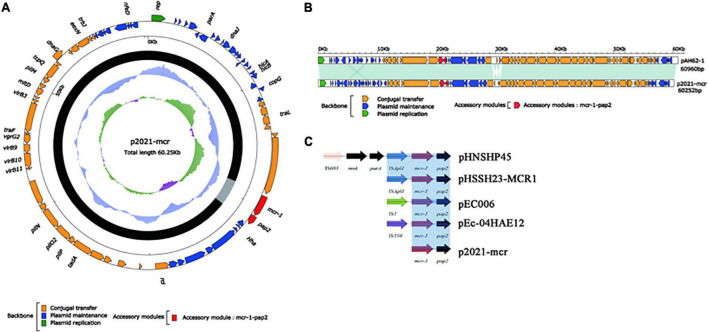
The complete sequence circular diagram of p2021-*mcr*. **(A)** The innermost ring represents (G-C)/(G + C); the blue ring indicates GC content, and the outward depression indicates that the GC content is higher than the mean value. Black area in the outer circle represents the backbone area and gray area represents the accessory modules. The outermost circle shows the distribution of genes represented by colored arrows in the plasmid. **(B)** Linear comparison of p2021-*mcr* and pAH62-1. Shadows indicate nucleotide homology is greater than 95%. **(C)** The diversity of *mcr-1* upstream environment of *rci* in Inc2 plasmid.

The IncI2-type plasmid p2021-*mcr* was extracted from *E. coli* strain DB20D28, with a length of 60.2 kb, the content was 42.3%, and a total of 78 ORFs (Figure 9A). Its structure was relatively simple as the whole plasmid consisted of a backbone region and a single *mcr-1-pap2. mcr-1* was the only ARG in p2021-*mcr*. The conjugation transfer region accounted for a large proportion of the plasmid, including flagellum formation gene *pilP* and conjugation transfer regulation gene *virB9*. Among them, the Shufflon DNA-specific recombinase secreted by the *rci* gene in this structure is an essential member of the recombinant system of the IncI plasmid, which obtained exogenous DNA through specific recombination. While there were no MGEs such as IS, Tn, and integron in p2021-*mcr*, *mcr-1* was the only ARG. IncI2 plasmids have a high overall structural similarity, while most of the difference is caused by gene-specific recombination upstream and downstream of *rci* (Gyohda et al., 2002). Linear comparison between p2021-*mcr* and reference plasmid pAH62-1 (Genbank: CP055260.1) (Figure 9B) showed that *mcr-1-pap2* structure also existed upstream of *rci* and the main difference was that partial sequence inversion occurred in the downstream of this gene due to recombination (Figure 9C).

### Analysis of multidrug-resistant region in p20432-*mcr*

**FIGURE 10:**
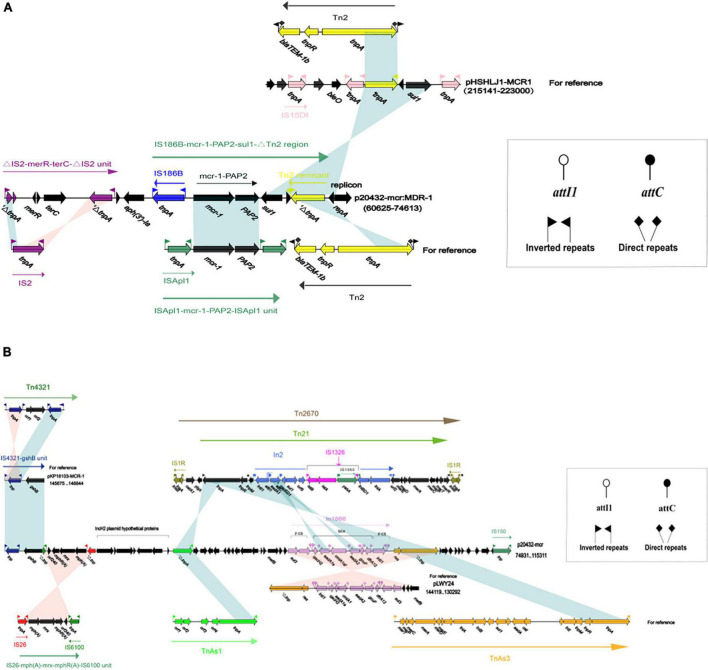
The organization and alignment of MDR-1 region of p20432-*mcr*. **(A)** Different colors indicate different moving elements, and arrows indicate genes. Shading indicates that the homology of the two parts of nucleic acids is greater than 95%, and the number in parentheses indicates the location of the fragment in the plasmid. **(B)** The organization and alignment of MDR-2 region and In*1866*-related region of p20432-*mcr*. Different colors indicate different moving elements, and arrows indicate genes. Shading indicates that the homology of the two parts of nucleic acids is greater than 95%, and the number in parentheses indicates the organization and alignment of MDR-2 region and In*1866*-related region of p20432-*mcr*.

The MDR-1 region of p20432-*mcr* is composed of MGEs and ARGs in order: △*IS2-merR-terC-△IS2-aph(3′)-Ia-IS186B-mcr-1-PAP2-1-sul-△Tn2*. *mcr-1* was followed by a PAP2 family of proteins, and *mcr-1*-pap2 has been reported in many plasmids, most of which were related to ISApl1. However, in p20432-*mcr*, IS*Apl1* has been replaced by IS*186B*. IS*186B-mcr-1-*PAP2 has no similar sequence in Genebank, but IS*186B* can transpose *mcr-1* into chromosomes (Saisree et al., 2001), promoting the vertical transmission of polymyxin resistance genes (Figure 10A). The multidrug -resistance region MDR-2 of the following mobile element in sequence IS*4321*, IS*6100*, IS*26*, Tn*Asl. IS26-mph(A)-mrx-mphR(A)-IS6100* is a classic antimicrobial resistance locus that contains a macrolide resistance gene *mph(A)* and a regulator *mphR*(A). A new type 1 integron was found and the number In*1866* was assigned by INTEGRALL database. The ARGs in gene cassette of In*1866* contained *dfr*A12 (Trimethoprim), *aad*A2 (aminoglycoside), *cmlAlaf* (chloramphenicol), *aad*A1 (aminoglycoside) and the 3 prime-CS region contained *sul3* (sulfanilamide) (Figure 10B). It is worth noting that the chloramphenicol resistance gene *cmlA1af* is a new variant of *cml*A1, which was first discovered in the gene cassette. The main difference is that the final two amino acids of the product encoded by *cmlA1af* changed from arginine—valine to serine—methionine.

## Discussion

China has the highest use of antimicrobials in husbandry each year and there was a long period that antimicrobials (e.g., C, TET, AMP, TMP) were used to treat bacterial infections or as antimicrobial growth promoters (Niu et al., 2020). As a result, selection for resistant bacteria has promoted gene mutation, recombination, and horizontal gene transfer in poultry farming environments, leading to the prevalence of multidrug-resistant bacteria. China is the largest producer and consumer of eggs globally and the second only to the United States in chicken production (Wang et al., 2015). The enormous and still increasing demands for poultry products in China have meant plenty of room to expand the production of layer and broiler poultry farms (Van Boeckel et al., 2015). Furthermore, the family poultry pattern will still be the primary choice for rural farmers in China. Identifying the phenotype and genotype of MDRE isolated from BFs and LFs indicated that different treatment methods and culture cycles could cause antimicrobial resistance in the environment and different ARGs. Several studies have confirmed that animals and the food chain play critical roles in the emergence and spreading of antimicrobial-resistant bacteria and ARGs (Luo et al., 2020; Tartor et al., 2021). Based on the “One Health” concept proposed by WHO, a coordinated, multisectoral approach is needed to address this complex problem of antimicrobial resistance in the environment, human health and animal husbandry (McEwen and Collignon, 2018). However, the close spatial distribution of residential and farm areas in the family poultry pattern makes the environment, human health and animal husbandry greatly influenced by each other. Thus, surveillance and research are essential to identify critical resistance points and prevent them. As the surveillance of the antimicrobial is used in both humans and non-humans, it is also necessary to implement differentiated and targeted surveillance strategies in broiler and layer poultry farms (Sahoo et al., 2010). Understanding of the differences in antimicrobial resistance profiles between broilers and layers and how they spread can help make effective antimicrobial resistance strategies in BFs and LFs.

This study demonstrated a high prevalence of MDR *Enterobacteriaceae* and high resistance-gene diversity in poultry farms. By identifying the resistance phenotype of MDRE, the results showed that tetracycline, sulfonamides, amoxicillin, and macrolides antimicrobials all had high resistance rates in both types of farms. The comparison of MDRE in BFs and LFs showed that the risk of MDRE isolated from BFs was higher than those from LFs. It is mainly reflected in two aspects: (1) multidrug-resistance rates were higher in BFs samples; (2) in terms of meropenem, amoxicillin, gentamicin, ciprofloxacin, trimethoprim-sulfamethoxazole, enrofloxacin, ofloxacin, and polymyxin B, the resistance rates of MDRE isolated from BFs were higher than LFs. These results may be caused by: (1) short breeding period and weak resistance of broilers; (2) broilers have a higher range of activities in comparison with the caged laying chickens, and therefore they were more susceptible to MDR infection; (3) some antimicrobials are forbidden in laying chicken breeding or during the laying period, but not as strict in broilers. The resistance rates of amoxicillin, trimethoprim, tetracycline, and sulfamethoxazole used in this study have reached more than 80%, while the resistance rates of polymyxin B and meropenem were lower than 20%. Previous studies detected polymyxin resistance in 40% of bacterial isolates from poultry samples, much higher than what was detected in our study (Lay et al., 2021). However, significant differences between broiler and layer samples (*P* ≤ 0.05) suggest different transmission characteristics of *mcr* genes in the two poultry farms. In addition, most of the polymyxin-resistant broiler sources MDRE were resistant to imipenem, gentamicin, ceftazidime, meropenem, cefoxitin, and norfloxacin. Moreover, relatively similar patterns were reported for *E. coli* against antimicrobials in Croatia and Egypt (Singh et al., 2018; Tartor et al., 2021). The polymyxin-resistant layer sources of MDRE were mainly resistant to imipenem, gentamicin, cefazolin, chloramphenicol, amoxicillin and tetracycline, which was inconsistent with other studies. Resistance of ESBL-producing *Enterobacteriaceae* to penicillins, cephalosporins and monobactams may force the use of last-resort antimicrobials as carbapenems and colistin (Elmonir et al., 2021). According to PCR results, *bla*_*TEM*_ was the predominant β-lactamase gene in both BFs (79.99%) and LFs (78.25%), but *bla*_*SHV*_ was not detected. In contrast, all *K. pneumoniae* isolates from broilers and their environment carried *bla*_*SHV*–2_ in Germany (Daehre et al., 2018). However, in Indonesia, *bla*_*TEM*_ (100%) was detected in all *K. pneumoniae* isolates of chicken origin and only 9.1% of the isolates carried *bla*_*SHV*_ showing a similar pattern to the results of this study (Hayati et al., 2019).

Since the plasmid carrying *mcr-1* was first reported in China in 2015, colistin resistance has spread rapidly worldwide, and the resulting development of *Enterobacteriaceae* from extensive drug resistance to pan-drug resistance is inevitable and will ultimately become global (Kumarasamy et al., 2010; Liu et al., 2016). A study based on the prevalence of *mcr-1* in the human intestine showed that a decline in colistin-resistant bacteria isolated from both animals and humans decreased significantly after the implementation of the colistin prohibition policy in China in 2017 (Wang et al., 2020; Pyw et al., 2020). It is agreed that the *mcr-1* is one of the few clear examples of animal origin resistance traits that can hit the entire human health system (Poirel and Nordmann, 2016). In this study, two *mcr-1* positive plasmids were found to have high *in vitro* transfer rates between *E. coli*, which are key human pathogens in broilers and layers. In particular, p20432-*mcr*, found in broiler MDRE, carries a novel integron In*1866* and multiple drug resistance genes. The emergence of In*1866* confirms that selection pressure is an important condition for the generation of new mobile components (Che et al., 2022). However, it remains to be studied how MGEs evolve through high-frequency HGT in a particular pattern of the family farms. The plasmid p20432-*mcr* isolated from broiler source *E. coli* RB20432 belongs to a complex chimeric plasmid, which likely contains both IncHI2 type and IncN type replicons. In addition, the structure of multiple replicons is conducive to increasing the host range of this plasmid, which is likely to make *mcr-1* spread more widely (Pilla and Tang, 2018). In a previous study, *E. coli* isolate possessed multiple resistance genes to β-lactams, aminoglycosides, tetracyclines, sulfonamides, and macrolides, which might be attributed to the presence IncFII, IncHI2, IncHI2A, and IncI1α replicon types (Hayati et al., 2019). In addition to polymyxin resistance genes, ARGs of chloramphenicol, aminoglycoside, and metal resistance genes carried by p20432-*mcr* make the plasmid transfer under a variety of selective pressures and the family poultry pattern is characterized by a greatly increased risk of transmission to humans. This problem will be more directly reflected in the health examination of the farmers in family farms. So, strategies for preventing the spread of antimicrobials resistance in such farming patterns need to be further improved.

## Conclusion

We conducted a comprehensive analysis of MDRE from typical broiler and laying farms in Zhejiang Province, China. The results showed that antimicrobials prohibition had a positive effect on reducing resistance genomes in the breeding environments. The detection rate of some ARGs banned in layer farms that were not suppressed in broilers was significantly increased in broiler source MDRE (*P* ≤ 0.05). However, our study showed that although certain antimicrobials such as polymyxin have been banned in poultry farming, the polymyxin resistance gene *mcr-1* persists in the environment for a long time due to spreading mobile elements like plasmids. The multidrug-resistant plasmid p20432-*mcr* analysis showed that the multi-replicon structure enabled it to transmit *mcr-1* and other ARGs to different hosts, resulting in a higher detection rate for *mcr-1*, *aph(3′)-Ia* and *aad*A2 in broiler farms. Therefore, it is necessary to implement and improve cleaning and disinfection procedures in family broiler farms and detect resistant bacteria from broilers and the environment to prevent further transmission to humans through the poultry production chain. It is also necessary to assess the antimicrobial resistance of bacteria from farmers on family farms.

## Data availability statement

The datasets presented in this study can be found in online repositories. The names of the repository/repositories and accession number(s) can be found below: https://www.ncbi.nlm.nih.gov/, PRJNA798875.

## Author contributions

SZ analyzed the data and wrote the manuscript. DX and XZ analyzed the data and prepared figures. DZ contributed in review and editing. PW collected samples and helped with software. DQ conceived the study and revised the original manuscript. All authors edited the manuscript and approved the final draft.

## Footnotes

1
http://www.heatmapper.ca/
