# Supplementary material for: The diversity in antimicrobial resistance of MDR Enterobacteriaceae among Chinese broiler and laying farms and two mcr-1 positive plasmids revealed their resistance-transmission risk
Source: Front Microbiol. 2022 Aug 4;13:912652. doi: 10.3389/fmicb.2022.912652 (PMC9387725; doi:10.3389/fmicb.2022.912652)

Supplement Figures

Figure S1 The detection rate of antibiotic resistance genes which are not in layers and broilers. (A) The detection rate of fosfomycin resistance genes. (B) The detection rate of chloramphenicol resistance genes.

(A)


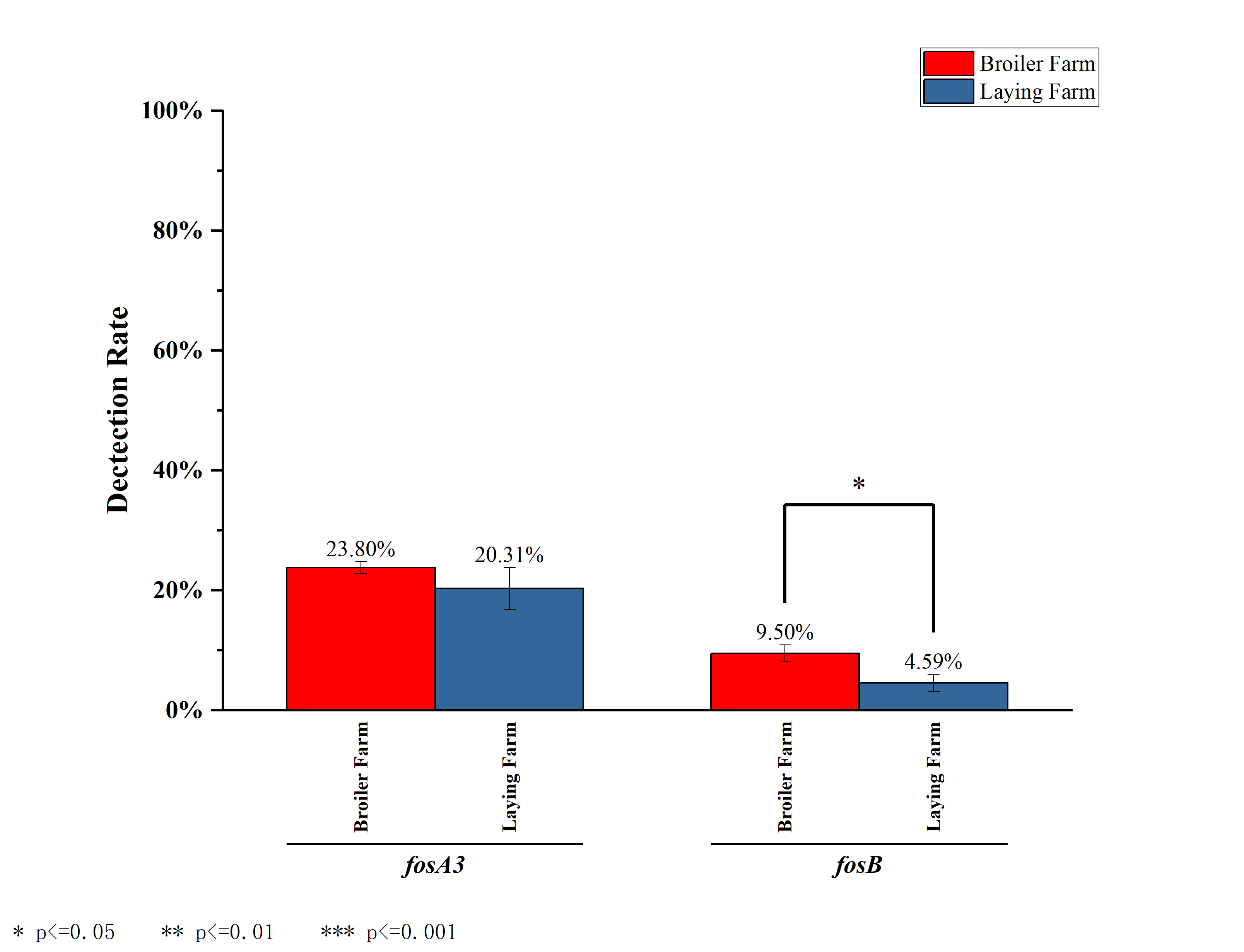
(B)


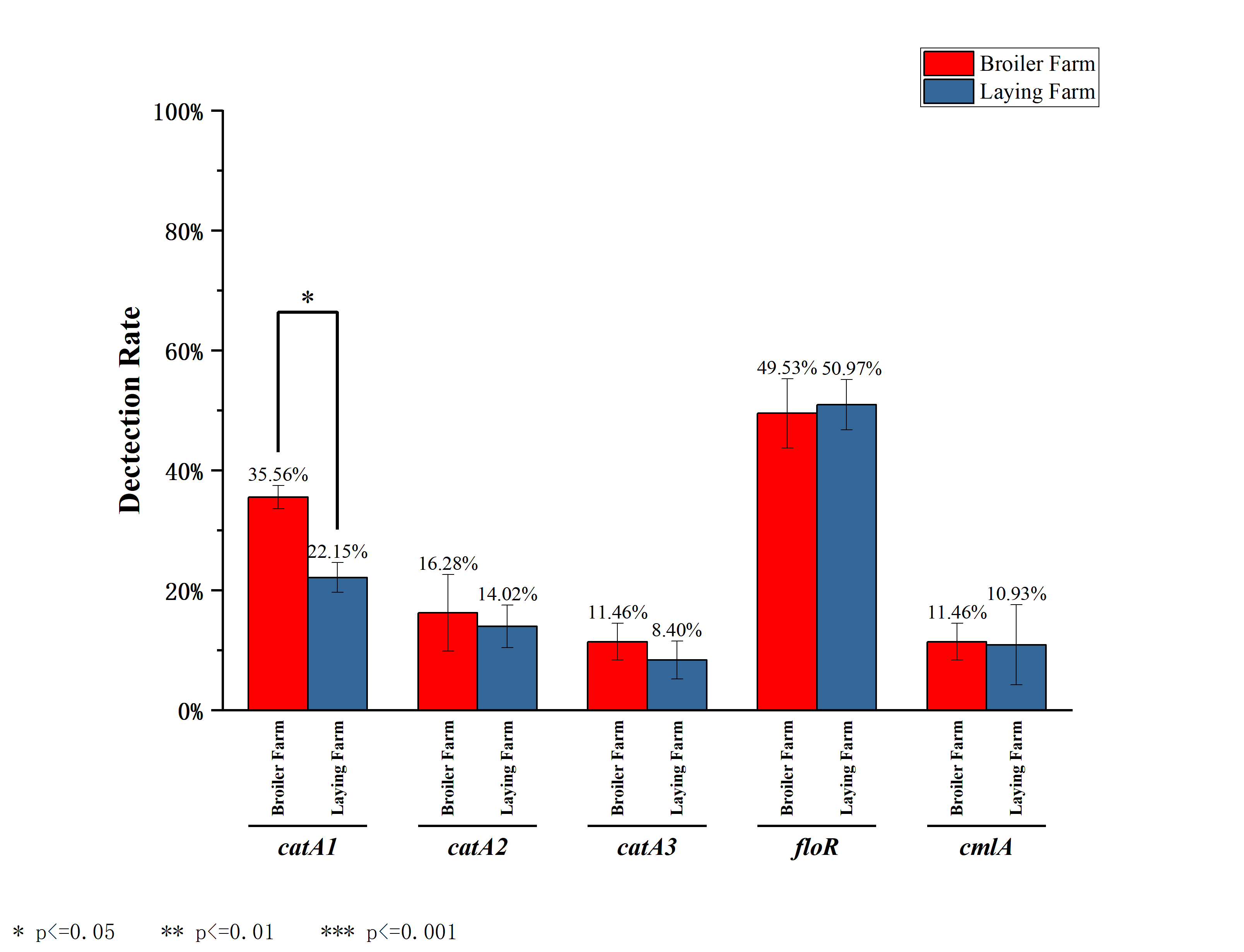

Supplement: Supplementary file 1 [file Table_1.DOCX]
