# Supplementary material for: The diversity in antimicrobial resistance of MDR Enterobacteriaceae among Chinese broiler and laying farms and two mcr-1 positive plasmids revealed their resistance-transmission risk
Source: Front Microbiol. 2022 Aug 4;13:912652. doi: 10.3389/fmicb.2022.912652 (PMC9387725; doi:10.3389/fmicb.2022.912652)
Supplement: Supplementary file 2 [file Table_2.DOCX]

**Table S1 Resistant gene primers used in this study**

| Antimicrobials | | Abbreviation | Breakpoint (μg/ml)  (CLSI, 2020) | | |
| --- | --- | --- | --- | --- | --- |
|  |  |  | S | I | R |
| β-lactams |  | AMX | ≤8 | 16 | ≥32 |
|  | Cefazolin | CZ | ≤16 | - | ≥328 |
|  | Ceftazidime | CAZ | ≤4 | 8 | ≥16 |
|  | Cefoxitin | FOX | ≤6 | 16 | ≥32 |
|  | Imipenem | IPM | ≤1 | 2 | ≥4 |
|  | Meropenem | MEM | ≤1 | 2 | ≥4 |
|  | Aztreonam | AT | ≤16 | - | ≥32 |
| Aminoglycosides | Amikacin | AK | ≤16 | 32 | ≥64 |
|  | Kanamycin | KAN | ≤16 | 32 | ≥64 |
|  | Gentamicin | GM | ≤4 | 8 | ≥16 |
| Quinolones | Ciprofloxacin | CIP | ≤0.06 | 0.12 | ≥1 |
|  | Norfloxacin | NOR | ≤4 | 8 | ≥16 |
|  | Ofloxacin | OFX | ≤0.12 | 0.25 | ≥2 |
| Sulfonamides | Trimethoprim | TMP | ≤8 | - | ≥16 |
|  | Sulfamethoxazole | SIZ | ≤256 | 13-16 | ≥512 |
|  | Trimethoprim-Sulfamethoxazole | SXT | ≤38 | 11-13 | ≥76 |
| Amphenicols | Chloramphenicol | C | ≤8 | 16 | ≥32 |
| Fosfomycin | Fosfomycin | FOS | ≤64 | 128 | ≥256 |
| AmoxicillinMacrolides | Azithromycin | AZM | ≤16 | - | ≥23 |
| Polymyxins | Polymyxin B | PB | ≤2 | - | ≥4 |
| Tetracyclines | Tetracycline | TE | ≤4 | 8 | ≥16 |
|  | Minocycline | MI | ≤4 | 8 | ≥16 |
