# Supplementary material for: The diversity in antimicrobial resistance of MDR Enterobacteriaceae among Chinese broiler and laying farms and two mcr-1 positive plasmids revealed their resistance-transmission risk
Source: Front Microbiol. 2022 Aug 4;13:912652. doi: 10.3389/fmicb.2022.912652 (PMC9387725; doi:10.3389/fmicb.2022.912652)
Supplement: Supplementary file 3 [file Table_3.DOCX]

**Table S2 Resistant gene primers used in this study**

| Target  Gene | Sequence(5’ -3’) | Amplicon length (bp) | Annealing Temperature (℃) | Reference |
| --- | --- | --- | --- | --- |
| 16s rRNA | 27F: YMAGAGTTTGATYMTGGCTCAG  1492R: TACCTTGTTACGACTT | 1492 | 50 | / |
| *bla_NDM-1_* | F: GGTTTGGCGATCTGGTTTTC  R: CGGAATGGCTCATCACGATC | 621 | 56 | ^1^ |
| *bla_KPC_* | F: GTATCGCCGTCTAGTTCTGC  R: GGTCGTGTTTCCCTTTAGCC | 638 | 56 | ^2^ |
| *bla_SME_* | F: GAGGAAGACTTTGATGGGAGGAT  R: TCCCCTCAGGACCGCCAAG | 334 | 52 | ^3^ |
| *bla_IMP_* | F: GGAATAGAGTGGCTTAAYTCTC  R: uAAAACAACCACC | 232 | 56 | ^4^ |
| *bla_OXA-23_* | F: GATCGGATTGGAGAACCAGA  R: ATTTCTGACCGCATTTCCAT | 501 | 56 | ^5^ |
| *bla_CTX-M_* | F: ATGTGCAGYACCAGTAARGT  R: TGGGTRAARTARGTSACCAGA | 593 | 52 | ^6^ |
| *bla_TEM_* | F: CATTTCCGTGTCGCCCTTATTC  R: CGTTCATCCATAGTTGCCTGAC | 800 | 52 | ^7^ |
| *bla_SHV_* | F: AGCCGCTTGAGCAAATTAAAC  R: ATCCCGCAGATAAATCACCAC | 713 | 52 |  |
| *bla_OXA-1 group_* | F: GGCACCAGATTCAACTTTCAAG  R: GACCCCAAGTTTCCTGTAAGTG | 564 | 52 |  |
| *qnrA* | F:CAGCAAGAGGATTTCTCACG  R:AATCCGGCAGCACTATTACTC | 630 | 57 | ^8^ |
| *qnrB* | F:GGCTGTCAGTTCTATGATCG  R:SAKCAACGATGCCTGGTAG | 488 | 57 |  |
| *qnrC* | F:GCAGAATTCAGGGGTGTGAT  R:AACTGCTCCAAAAGCTGCTC | 118 | 57 |  |
| *qnrD* | F:CGAGATCAATTTACGGGGAATA  R:AACAAGCTGAAGCGCCTG | 581 | 57 |  |
| *qnrS* | F:GCAAGTTCATTGAACAGGGT  R:TCTAAACCGTCGAGTTCGGCG | 428 | 57 |  |
| *qnrVC* | F:GGATAAAACAGACCAGTTATATGTACAAG  R:AGATTTGCGCCAATCCATCTATT | 444 | 57 |  |
| *oqxAB* | F:CCGCACCGATAAATTAGTCC  R:GGCGAGGTTTTGATAGTGGA | 313 | 57 |  |
| *qeqA* | F:GCAGGTCCAGCAGCGGGTAG  R:CTTCCTGCCCGAGTATCGTG | 218 | 57 |  |
| *mph(A)* | F:GTGAGGAGGAGCTTCGCGAG  R:TGCCGCAGGACTCGGAGGTC | 403 | 56 | ^9^ |
| *mph(B)* | F:GATATTAAACAAGTAATCAGAATAG  R:GCTCTTACTGCATCCATACG | 494 | 56 |  |
| *mph(D)* | F:AGCCAATTGCTACATGCGCTCT  R:GGGTTTACGAGCCAAGCAAGAA | 756 | 56 |  |
| *mph(E)* | F:ATGCCCAGCATATAAATCGC  R:ATATGGACAAAGATAGCCCG | 271 | 56 |  |
| *erm(A)* | F:TCTAAAAAGCATGTAAAAGAAA  R:CGATACTTTTTGTAGTCCTTC | 533 | 56 |  |
| *erm(B)* | F:GAAAAAGTACTCAACCAAATA  R:AATTTAAGTACCGTTACT | 639 | 45 |  |
| *erm(C)* | F:TCAAAACATAATATAGATAAA  R:GCTAATATTGTTTAAATCGTCAAT | 642 | 45 |  |
| *tetA* | F:GCTACATCCTGCTTGCCTTC  R:CATAGATCGCCGTGAAGAGG | 210 | 50 | ^10^ |
| *tetB* | F:TTGGTTAGGGGCAAGTTTTG  R:GTAATGGGCCAATAACACCG | 659 | 50 |  |
| *tetC* | F:CTTGAGAGCCTTCAACCCAG  R:ATGGTCGTCATCTACCTGCC | 418 | 49 |  |
| *tetD* | F:AAACCATTACGGCATTCTGC  R:GACCGGATACACCATCCATC | 787 | 48 |  |
| *catA1* | F:GGGTGAGTTTCACCAGTTTTGATT  R: CACCTTGTCGCCTTGCGTATA | 952 | 52 | ^11^ |
| *catA2* | F: GCACTCGATGCCTTCCAAAA  R: AGAGCCGATCCAAACGTCAT | 482 | 56 |  |
| *catA3* | F:AGGAAGCATCGGAACGTTGA  R:ACAGACCGAGCACGACTGTTG | 358 | 52 |  |
| *floR* | F:ATTGTCTTCACGGTGTCCGTTA  R:CCGCGATGTCGTCGAACT | 962 | 52 |  |
| *cmlA* | F:TAGGAAGCATCGGAACGTTGAT  R:CAGACCGAGCACGACTGTTG | 665 | 56 |  |
| *fosA3* | F:ATTCCCCACATGCAGCTCCAGCTTATGGCC  R:AAACGCCCCCTCAGGAAAGCGACTATACCC | 182 | 53 | ^12^ |
| *fosB* | F:TCACTGTAACTAATGAAGCATTAGACCAT  R:CCATCTGGATCTGTAAAGTAAAGAGATC | 271 | 60 |  |
| *fosX* | F:GATTAAGCCATATCACTTTAATTGTGAAAG  R:TCTCCTTCCATAATGCAAATCCA | 217 | 50 |  |
| *aphA6* | F: ATACAGAGACCACCATACAGT  R: GGACAATCAATAATAGCAAT | 235 | 55 | ^13^ |
| *aacC* | F:CGTCACTTATTCGATGCCCTTAC  R:GTCGGGCGCGGCATA | 465 | 57 |  |
| *aadA2* | F:ACGGCTCCGCAGTGGAT  R:GGCCACAGTAACCAACAAATCA | 265 | 56 |  |
| *aac(6')-Ib-cr* | F:TTGGAAGCGGGGACGGAM  R:ACACGGCTGGACCATA | 260 | 57 |  |
| *sul1* | F:TCATCTGCCAAACTCGTCGTTA  R:GTCAAAGAACGCCGCAATGT | 105 | 56 | ^14^ |
| *sul2* | F:CAGCGCTATGCGCTCAAG  R:ATCCCGCTGCGCTGAGT | 129 | 56 |  |
| *folp1* | F:CAGGCTCGTAAATTGATAGCAGAAG  R:CTTTCCTTGCGAATCGCTTT | 140 | 55 |  |
| *folp2* | F:GCGATTCGCAAGGAAAGTGA  R:CACATGGGCCATTTTTTCATC | 138 | 54 |  |
| *folp3* | F:CACGGCTTCGGCTCATGT  R:TGCCATCCTGTGACTAGCTACGT | 288 | 58 |  |
| *mcr-1* | F:CGGTCAGTCCGTTTGTTC  R:CTTGGTCGGTCTGTAGGG | 309 | 56 | ^15^ |
| *mcr-2* | F:TGTTGCTTGTGCCGATTGGA  R:CAGCAACCAACAATACCATCT | 567 | 60 | ^16^ |
| *mcr-3* | F:AGTTTGGTTTCGCCATTTCATTAC  R:ATATCACTGCGTGGACAGTCAGG | 1084 | 58 |  |
| *mcr-4* | F:TTACAGCCAGAATCATTATCA  R:ATTGGGATAGTCGCCTTTTT | 488 | 58 |  |
| *intI1* | F:GGCTTCGTGATGCCTGCTT  R:CATTCCTGGCCGTGGTTCT | 149 |  | ^17^ |
| *intI2* | intI2L:CACGGATATGCGACAAAAAGGT  Int2R:GTAGCAAACGAGTGACGAAATG | 789 |  | ^18^ |
| *intI3* | int3L:GCCTCCGGCAGCGACTTTCAG  Int3R:ACGGATCTGCCAAACCTGACT | 980 |  |  |
| Cassette arrays in class 1 integrons | hep58:TCATGGCTTGTTATGACTGT  hep59:GTAGGGCTTATTATGCACGC | class 1 integron variable region |  | ^19^ |
| Cassette arrays in class 2 integrons | hep51: GATGCCATCGCAAGTACGAG  hep74: CGGGATCCCGGACGGCATGCACGATTTGTA | class 2 integron variable region |  |  |

**Reference:**

1 Sang, S. *et al.* Multiplex PCR for rapid detection of genes encoding class A carbapenemases. *Annals of laboratory medicine* (2012).

2 Hong, S. S. *et al.* Multiplex PCR for rapid detection of genes encoding class A carbapenemases. *Annals of Laboratory Medicine* **32**, 359 (2012).

3 Voets, G. M., Fluit, A., Scharringa, J., Stuart, J. C. & Leverstein-van Hall, M. A. A set of multiplex PCRs for genotypic detection of extended-spectrum β-lactamases, carbapenemases, plasmid-mediated AmpC β-lactamases and OXA β-lactamases. *International journal of antimicrobial agents* **37**, 356-359 (2011).

4 Poirel, L., Walsh, T. R., Cuvillier, V. & Nordmann, P. Multiplex PCR for detection of acquired carbapenemase genes. *Diagnostic microbiology and infectious disease* **70**, 119-123 (2011).

5 Woodford, N. *et al.* Multiplex PCR for genes encoding prevalent OXA carbapenemases in Acinetobacter spp. *International journal of antimicrobial agents* **27**, 351-353 (2006).

6 Pagain, L. *et al.* Multiple CTX-M type extended-spectrum B-lactamases in nosocomonal isolates of Enterobacteriaceacea from hospital in North Italy. *J Clin Microbial* **41**, 4264-4269 (2003).

7 Dallenne, C., Da Costa, A., Decré, D., Favier, C. & Arlet, G. Development of a set of multiplex PCR assays for the detection of genes encoding important β-lactamases in Enterobacteriaceae. *Journal of Antimicrobial Chemotherapy* **65**, 490-495 (2010).

8 Ciesielczuk, H., Hornsey, M., Choi, V., Woodford, N. & Wareham, D. Development and evaluation of a multiplex PCR for eight plasmid-mediated quinolone-resistance determinants. *Journal of Medical Microbiology* **62**, 1823-1827 (2013).

9 Nguyen, M. C. P. *et al.* Escherichia coli as reservoir for macrolide resistance genes. *Emerging infectious diseases* **15**, 1648 (2009).

10 Seifi, S. & Khoshbakht, R. Prevalence of tetracycline resistance determinants in broiler isolated Escherichia coli in Iran. *British poultry science* **57**, 729-733 (2016).

11 Du, Z. *et al.* The prevalence of amphenicol resistance in Escherichia coli isolated from pigs in mainland China from 2000 to 2018: A systematic review and meta-analysis. *PloS one* **15**, e0228388 (2020).

12 Wachino, J.-i., Yamane, K., Suzuki, S., Kimura, K. & Arakawa, Y. Prevalence of fosfomycin resistance among CTX-M-producing Escherichia coli clinical isolates in Japan and identification of novel plasmid-mediated fosfomycin-modifying enzymes. *Antimicrobial Agents and Chemotherapy* **54**, 3061-3064 (2010).

13 Vila, J. *et al.* Spread of amikacin resistance in Acinetobacter baumannii strains isolated in Spain due to an epidemic strain. *Journal of clinical microbiology* **37**, 758-761 (1999).

14 Rolbiecki, D., Harnisz, M., Korzeniewska, E., Jałowiecki, Ł. & Płaza, G. Occurrence of Fluoroquinolones and Sulfonamides Resistance Genes in Wastewater and Sludge at Different Stages of Wastewater Treatment: A Preliminary Case Study. *Applied Sciences* **10**, 5816 (2020).

15 Wei, P. *et al.* Substrate analog interaction with MCR‐1 offers insight into the rising threat of the plasmid‐mediated transferable colistin resistance. *The FASEB Journal* **32**, 1085-1098 (2018).

16 Xavier, B. B. *et al.* Identification of a novel plasmid-mediated colistin-resistance gene, mcr-2, in Escherichia coli, Belgium, June 2016. *Eurosurveillance* **21**, 30280 (2016).

17 Luo, Y. *et al.* Trends in antibiotic resistance genes occurrence in the Haihe River, China. *Environmental science & technology* **44**, 7220-7225 (2010).

18 Odumosu, B. T., Adeniyi, B. A. & Chandra, R. Analysis of integrons and associated gene cassettes in clinical isolates of multidrug resistant Pseudomonas aeruginosa from Southwest Nigeria. *Annals of clinical microbiology and antimicrobials* **12**, 1-7 (2013).

19 White, P. A., McIver, C. J. & Rawlinson, W. D. Integrons and gene cassettes in the Enterobacteriaceae. *Antimicrobial agents and chemotherapy* **45**, 2658-2661 (2001).
