# Supplementary material for: The diversity in antimicrobial resistance of MDR Enterobacteriaceae among Chinese broiler and laying farms and two mcr-1 positive plasmids revealed their resistance-transmission risk
Source: Front Microbiol. 2022 Aug 4;13:912652. doi: 10.3389/fmicb.2022.912652 (PMC9387725; doi:10.3389/fmicb.2022.912652)
Supplement: Supplementary file 5 [file Data_Sheet_1.PDF]

UMR Inserm 1092
Anti-infectieux : supports moléculaires des résistances et innovations thérapeutiques

CBRS
Rue du Pr Bernard Descotttes
87 025 Limoges Cedex, France
Tél : (33) (0) 519 564 263
Fax : (33) (0) 555 435 851
Mél : thomas.jove@inserm.fr

Limoges, le 21st January 2022

Objet : In1866 integron certificate

To whom it may concern,

I, undersigned Thomas Jové, as a curator of the INTEGRALL database (http://integrall.bio.ua.pt/), hereby certify In1866 to be a class 1 integron with the *dfrA12/gcuF/aadA2/cmlA1af/aadA1a/qacF* structure. It has first been assigned to Pr Daofeng Qu (Zhejiang Gongshang University) whose group submitted the sequence in 2021.

Sincerely yours,

Thomas Jové, INTEGRALL curator
