# Supplementary figures and images for: The diversity in antimicrobial resistance of MDR Enterobacteriaceae among Chinese broiler and laying farms and two mcr-1 positive plasmids revealed their resistance-transmission risk
Source: Front Microbiol. 2022 Aug 4;13:912652. doi: 10.3389/fmicb.2022.912652 (PMC9387725; doi:10.3389/fmicb.2022.912652)

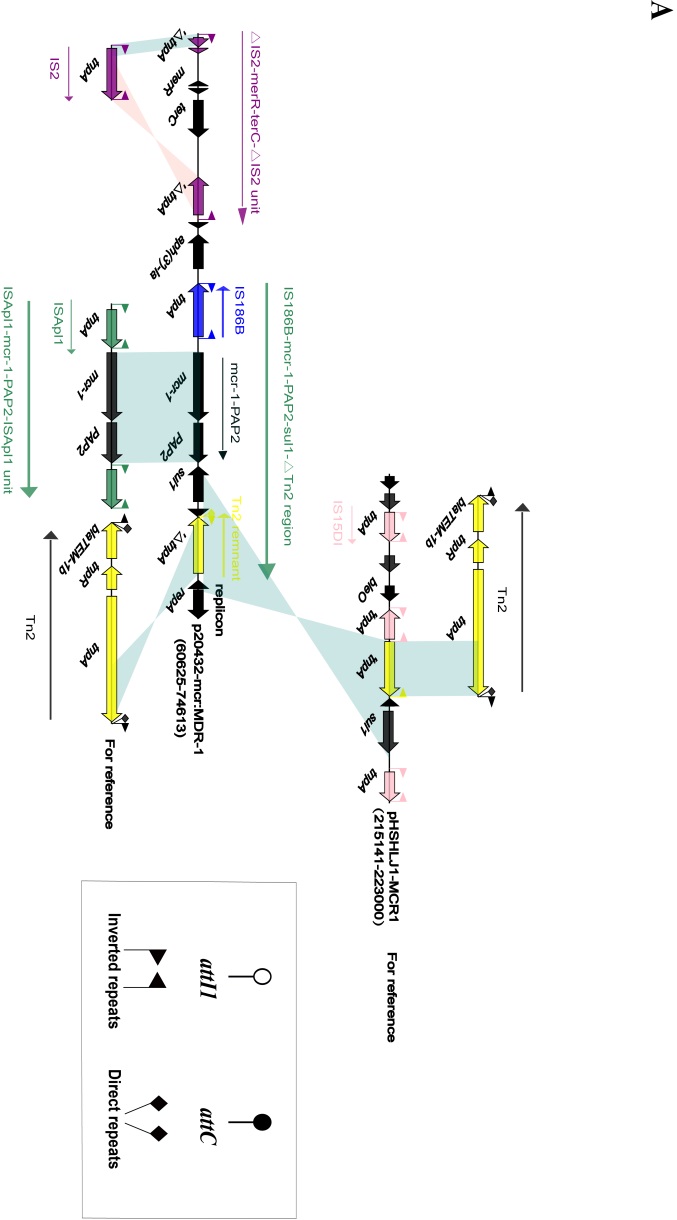

Supplement: Supplementary file 6 [file Image_1.JPEG]

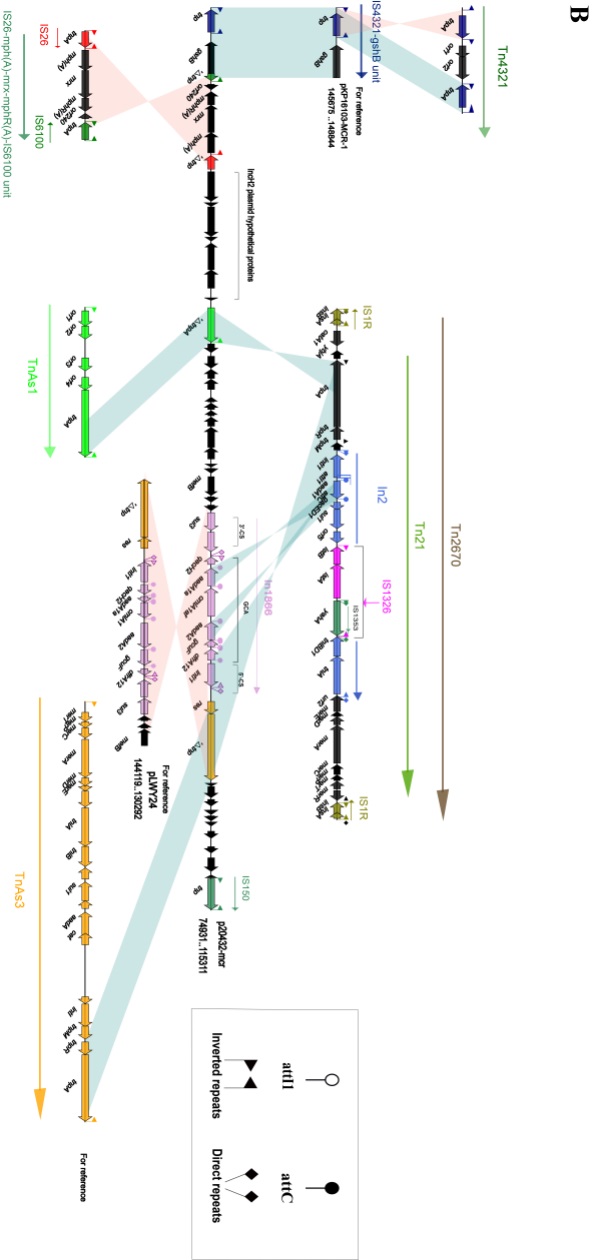

Supplement: Supplementary file 7 [file Image_2.JPEG]
